# Balancing Stability and Flexibility within the Genome of the Pathogen *Cryptococcus neoformans*

**DOI:** 10.1371/journal.ppat.1003764

**Published:** 2013-12-12

**Authors:** Kate L. Ormerod, James A. Fraser

**Affiliations:** 1 Australian Infectious Diseases Research Centre, University of Queensland, Brisbane, Queensland, Australia; 2 School of Chemistry and Molecular Biosciences, University of Queensland, Brisbane, Queensland, Australia; The University of North Carolina at Chapel Hill, United States of America

Over the last 30 years, the growing immunocompromised population has created fertile ground for opportunistic pathogens, particularly those from the Kingdom Fungi. While the range of fungal species causing infections is increasing, there remain three key culprits [1]. Firstly, the ascomycete yeast *Candida albicans* is responsible for the greatest number of fungal infections, particularly those acquired in a hospital setting. Secondly, the mould *Aspergillus fumigatus*, also an ascomycete, has become a cause of high mortality, particularly in transplant patients and those with hematological diseases. Finally, the basidiomycete yeast *Cryptococcus neoformans* has become a scourge of AIDS patients, accounting for an estimated 624,000 deaths per annum [2]. The genomes of *C. albicans* and *A. fumigatus* were published in 2004 [3] and 2005 [4] respectively, and the *C. neoformans* var. *neoformans* genome was published in 2005 [5]. Excitingly, the var. *grubii* genome, the variety responsible for the majority of infections [6], is nearing publication [G. Janbon, personal communication]. The analysis of these *C. neoformans* sequences, coupled with earlier karyotypic analyses, has uncovered a paradox: a pathogen with a highly stable genome that appears to have the capacity to undergo gross chromosomal rearrangement when required. This “flexible stability” could potentially represent an adaptive strategy supporting the opportunistic nature of this important pathogen.

## Early Insights into the *Cryptococcus* Genome

The earliest genomic studies in *C. neoformans* came from analysis of electrophoretic karyotype [7]. Surveys of the pathogen by John Perfect's group described variation in chromosome size and number between isolates, suggesting the genome was highly flexible and tolerant of rearrangement [8]. Extensive karyotyping by Teun Boekhout's laboratory followed, confirming the extent of the variability within the population and demonstrating a role for the sexual cycle in generating karyotypic diversity within the species [9], [10]. Excitingly, this early work suggested a link between genomic change and infection; in some instances, serial clinical isolates exhibited changes in chromosome sizes between infections, as did half of strains passaged through a mouse model of infection [11]. Such high frequency of variability strongly suggested these changes contribute to adaptability under selective pressure encountered in the host, consistent with the observation that these strains often exhibit changes in virulence-associated phenotypes [12], [13].

The nature of the gross chromosomal changes leading to such karyotypic diversity was extremely difficult to elucidate in the pregenomics era. Our first insights into *C. neoformans* chromosomal rearrangement came from study of the evolution of the mating-type (*MAT*) locus. *C. neoformans* has a bipolar mating-type system consisting of *MAT*α and *MAT* **a** alleles that encode sex-determining homeodomain transcription factors, pheromones, and pheromone receptors. Over a decades' work performed by several laboratories gradually revealed that in this pathogen the *MAT* locus is large. In comparison to the ∼2.5 kb of *S. cerevisiae* [14], early estimates of *MAT* size were between 35 and 75 kb [15], [16], and finally yielded sizes of 105 and 117 kb for var. *neoformans* α and **a** respectively, and 103 and 127 kb for var. *grubii* [17]. Phylogenetic and synteny analyses support the locus having evolved through a translocation bringing ancient homeodomain and pheromone/pheromone receptor loci together. Subsequent inversions, gene conversions and transposon accumulation resulted in a highly divergent gene order within the locus, in contrast with the synteny of the flanking regions [17], [18].

## *Cryptococcus* Enters the Genomic Era

The study of *MAT* provided the groundwork for understanding the *C. neoformans* genome and how it is evolving, the next stage of which began with the generation of linkage maps outlining the genomic architecture of the species [19], [20] and supporting the subsequent publication of the full genome sequence of *C. neoformans* var. *neoformans* in 2005 [5]. Sequencing of two related strains, JEC21 and B-3501A, revealed a 20 Mb haploid genome consisting of 14 chromosomes ranging in size from 762 kb to 2.3 Mb and each containing a regional centromere in the form of a transposon cluster. Over 6,500 genes were identified with an average size of 1.9 kb distributed over an average of 6.3 exons. Transposons represented ∼5% of the genome and clustered not only at regional centromeres but also adjacent to the rDNA repeats and within *MAT*.

During assembly, an anomaly was observed: only 13 chromosomes were evident for JEC21, one of which contained two transposon clusters rather than the single predicted centromeric clusters on the other 12. Interrogation of this unusual assembly by Joe Heitman's group revealed a unique genomic event [21]. It became apparent that during construction of the JEC20/JEC21 congenic laboratory pair, a telomere-telomere fusion had occurred followed by breakage of the dicentric intermediate and duplication of a 62 kb fragment containing 22 genes. While it is unknown whether this event was a result of a recombination between subtelomeric transposable elements or of nonhomologous end joining, it did further indicate a propensity for large-scale genomic events in *C. neoformans*.

The release of the genome for *C. neoformans* var. *neoformans*, as with all important species, heralded a proliferation of comparative genomic analyses. The initial comparison was with the var. *grubii* type strain H99, the genome of which had been available in draft format since 2001 as a collaboration between Duke University and the Broad Institute. The genomes of these closely related varieties share 85–90% sequence identity and are largely colinear [22], although this colinearity breaks down in the subtelomeric regions [23], centromeres, and *MAT*. In contrast to the early karyotypic analyses, such similarity supports a model of high genome stability since divergence of the varieties an estimated 18.5 million years ago [24].

During comparisons of var. *neoformans* and var. *grubii*, Fred Dietrich and colleagues discovered a 40 kb region containing 14 genes where the two varieties were almost identical (98.5% similar), which was dubbed the Identity Island [22]. Significantly this region was located on nonhomologous chromosomes: chromosome 6 in var. *neoformans* and 5 in var. *grubii*. Through comparison with *C. gattii*, it was determined that the Island had introgressed into var. *neoformans* from var. *grubii*, explaining the high nucleotide identity. Such an event could occur during formation of hybrid diploids [25]. Since the acquisition of the Identity Island the native copy of the region has been mostly eradicated in var. *neoformans*. Interestingly, JEC21 also contains a duplication of a 14 kb segment of the Identity Island.

**Figure 1:**
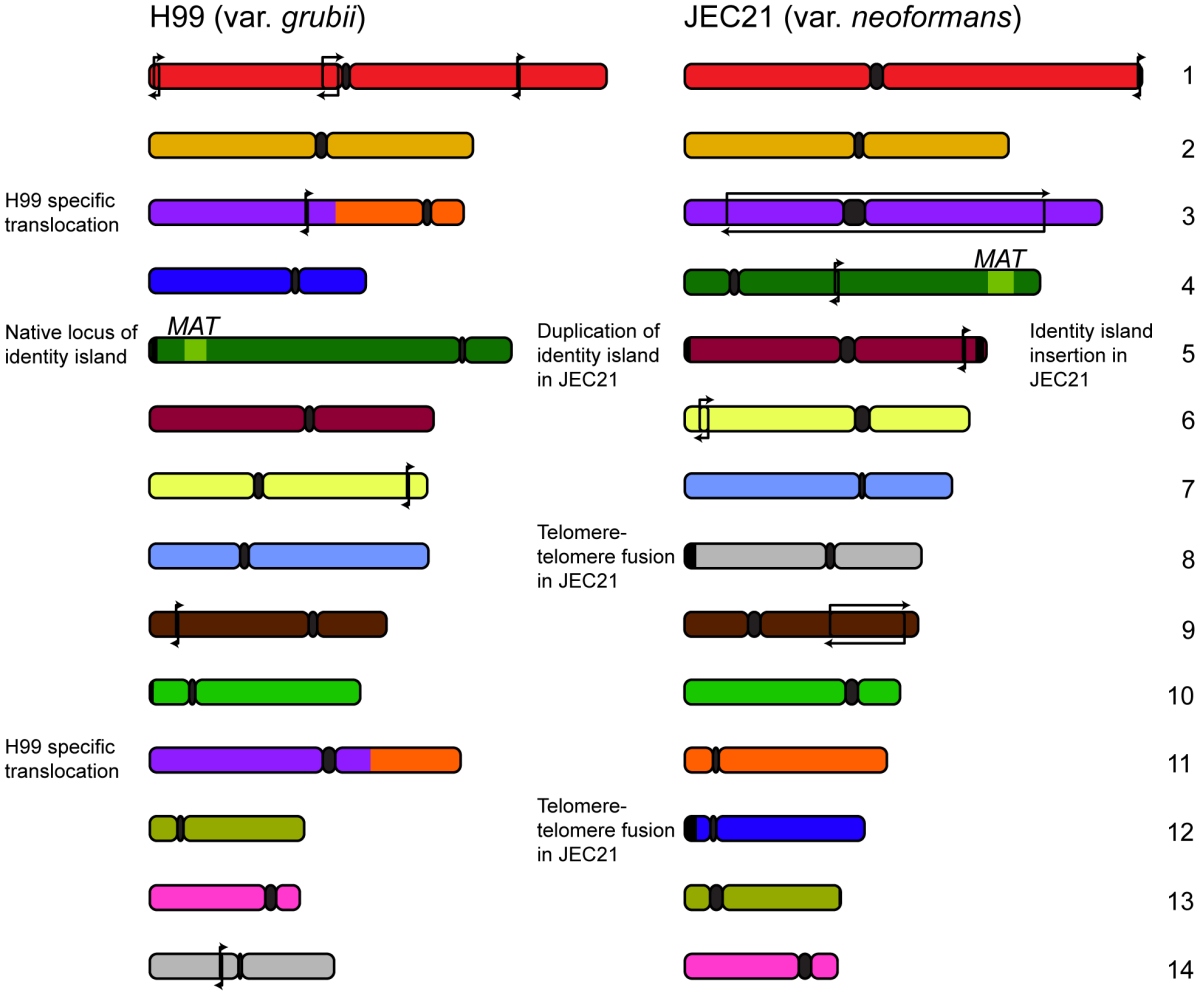
Genomic comparisons between var. *grubii* and var. *neoformans* reveal relatively few rearrangements. Since divergence 18.5 million years ago, the two varieties of *C. neoformans* have maintained a largely syntenic genome structure with only a few inversions specific to each variety (marked with arrows). Comparisons also uncovered several strain-specific events: a translocation in the case of the var. *grubii* type strain H99 and telomere-telomere fusion and introgression in the case of var. *neoformans* JEC21.

Further comparative studies by Sun and Xu revealed more insights into the evolutionary changes in these varieties' genomes, describing a number of small rearrangements [26]. The source of these changes was implied by the presence of transposable elements in association with around half of the events. Transposons are a principal source of genomic rearrangement in *S. cerevisiae* [27], a trend that appears to also hold true in *C. neoformans*. Further analysis conducted by our own group permitted the designation of each of the rearrangements occurring outside *MAT* or the centromeres as either var. *grubii* or var. *neoformans* specific [28] (Figure 1). Given the previously observed karyotypic variability, the relatively small number of changes fixed within the two varieties over millions of years is somewhat astonishing.

In addition to these small events, a translocation involving chromosomes 3 and 11 was found to be unique to the var. *grubii* type strain H99 [26]. Sequencing across the translocation breakpoint on chromosomes 3 and 11 identified a 3 bp microhomology consistent with the event arising via nonhomologous end joining [28]. Significantly, this sole karyotypically observable event was found within a clinically derived lineage, suggesting this type of selective pressure as a necessary precursor.

## Microevolution and Beyond

While these sequence-based studies relied on data from just a few strains, the advent of more high-throughput genomic technologies made larger-scale studies possible. Initially, comparative genomic hybridization (CGH) by Jim Kronstad and colleagues uncovered a significant number of previously unobserved amplifications and deletions in comparisons within both var. *neoformans* and var. *grubii* [29]. Most importantly, CGH uncovered a propensity for aneuploidy within *C. neoformans*, and this characteristic was found to be responsible for the intrinsic heteroresistance to the widely used antifungal fluconazole characterised by June Kwon-Chung's group [30], [31]. Resistant strains were found to have duplications of chromosome 1 (all strains) in addition to chromosomes 4, 10, and 14 in some strains [30]. Aneuploid strains were also found in freshly obtained clinical isolates and could be generated via passage through mice [32].

Key to the observations of karyotypic variability of clinical isolates, aneuploidy in association with heteroresistance and infection, and translocations specific to clinical lineages, is the application of selective pressure encountered in the host. This situation is similar to that seen in *C. albicans*, where genomic reorganization has been proposed as a mechanism of coping with selective pressure [33]. However, selective pressure is not only encountered in the host. What then is behind the overall stability of the genome? One possibility is a requirement for retention of a sexual cycle by the species. Spores produced as a result of matings enable dispersal in times of stress and are also infectious [34], [35]. Strains with genomes too far from the norm could be filtered out by this process either through sexual isolation, as seen in the separation of *C. gattii* [36], or through disruption of key genes required for mating. Increased genomic reorganization in asexual species such as *C. glabrata*, including the generation of novel chromosomes, supports this idea [37].

Sex in nature between opposite mating types of *C. neoformans* is known to occur in some populations [6]. However, the overwhelming global predominance of the α mating type of var. *grubii* means it is a parasexual cycle between isolates of the same mating type that is most relevant, and evidence of recombination within single-sex subpopulations supports its occurrence in the environment [38], [39]. Laboratory-passaged isolates often lose their ability to mate, indicating the requirement for selective pressure to maintain the process and therefore some associated advantage [40]. One advantage to a predominantly single-sex population could be the generation of novel diversity, as opposed to the mixing of existing diversity, recently demonstrated by Joe Heitman and colleagues to be a result of homothallic mating [41]. Thus sex not only permits dispersal but is a source of genome flexibility in closely related strains.

Genome resequencing projects are the next step toward fully understanding the balance between stability and flexibility within the genome of *C. neoformans*. This approach enables the detailed comparison of multiple isolates from a single patient and thus has the potential to fully assess the impact of long-term culture of *C. neoformans* in the human body on genomic variability. The first analysis of this type carried out by our laboratory compared two strains obtained from a female AIDS patient 77 days apart and uncovered aneuploidy of chromosome 12: two copies of the entire chromosome in the first isolate and one complete copy plus two additional copies of the left arm in the later isolate, observable as a mini-chromosome during pulsed-field gel electrophoresis [42]. The first isolate also contained a large inversion. Analysis at the nucleotide level revealed the isolates were separated by three SNPs and two indels, one of which leads to loss of a predicted transcriptional regulator causing changes in carbon source utilization and virulence. For the first time, this study provided evidence of large-scale plasticity between very closely related isolates. Now what is required is the confirmation of to what extent this flexibility is induced by the host environment.

The power of genome resequencing projects lies in their ability to be performed on an increasingly large scale. Bulk analysis of sequential clinical isolates will overcome the inevitable difficulties associated with dealing with the uncontrolled experimental environment of the human host and permit the designation of a typical level of genome flexibility that can be incorporated into the definition of mixed infection [43]. In addition, controlled mouse experiments coupled with sequencing will provide the required temporal data to associate genome changes with infection. The extensively curated annotations of var. *grubii*, now available to the community via the Broad Institute as a prelude to the highly anticipated genome paper, complete the foundation on which this future work will build.
